# Similar Multimorbidity Patterns in Primary Care Patients from Two European Regions: Results of a Factor Analysis

**DOI:** 10.1371/journal.pone.0100375

**Published:** 2014-06-23

**Authors:** Beatriz Poblador-Plou, Marjan van den Akker, Rein Vos, Amaia Calderón-Larrañaga, Job Metsemakers, Alexandra Prados-Torres

**Affiliations:** 1 EpiChron Research Group on Chronic Diseases, Aragón Health Sciences Institute (IACS), IIS Aragón, Miguel Servet University Hospital, Zaragoza, Spain; 2 Red de Investigación en Servicios de Salud en Enfermedades Crónicas (REDISSEC), Carlos III Health Institute, Madrid, Spain; 3 Teaching Unit of Preventive Medicine and Public Health, Aragón Health Sciences Institute (IACS), IIS Aragón, Zaragoza, Spain; 4 CAPHRI School for Public Health and Primary Care, Department of Family Medicine, Maastricht University, Maastricht, The Netherlands; 5 Department of General Practice, KU Leuven, Belgium; 6 Department of Microbiology, Preventive Medicine and Public Health, University of Zaragoza, Zaragoza, Spain; CUNY, United States of America

## Abstract

**Objective:**

To compare the similarities among the multimorbidity patterns identified in primary care patients from two European regions (Spain and the Netherlands) with similar organisational features of their primary care systems, using validated methodologies.

**Methodology:**

This observational, retrospective, multicentre study analysed information from primary care electronic medical records. Multimorbidity patterns were assessed using exploratory factor analysis of the diagnostic information of patients over 14 years of age. The analysis was stratified by age groups and sex.

**Results:**

The analysis of Dutch data revealed a higher prevalence of multimorbidity which corresponds with the clustering of a higher number of diseases in each of the patterns. Relevant clinical similarities were found between both countries for three multimorbidity patterns that were previously identified in the original Spanish study: cardiometabolic, mechanical and psychiatric-substance abuse. In addition, the clinical evolution towards complexity of the cardiometabolic pattern with advancing age -already demonstrated in the original study- was corroborated in the Dutch context. A clear association between mechanical and psychosocial disorders was unique to the Dutch population, as well as the recurrent presentation of the psychiatric-substance abuse pattern in all age and sex groups.

**Conclusions:**

The similarities found for the cardiometabolic, mechanical and psychiatric-substance abuse patterns in primary care patients from two different European countries could offer initial clues for the elaboration of clinical practice guidelines, if further evidenced in other contexts. This study also endorses the use of primary care electronic medical records for the epidemiologic characterization of multimorbidity.

## Introduction

Multimorbidity, which refers to the coexistence of two or more chronic conditions, is highly prevalent in older populations and, in absolute numbers, even more common in the adult population [1]. As a consequence, many people experience a decline of functional capacity and decreased quality of life, which leads to higher health care costs [2]–[4]. The negative outcomes associated with multimorbidity are largely attributable to the single-disease orientation of modern medicine [5], although diseases are rarely presented in isolation.

Lately, the global move towards the primary care-led management of chronic diseases has incorporated the analysis of multimorbidity patterns into the research agenda, with the goal of providing health and social care professionals with a better clinical and epidemiological understanding of the synergies and effects associated with coexisting diseases [6]. Studies aimed at classifying non-random associations between diseases into patterns and identifying the potential factors underlying such associations have emerged gradually in the last five years [7]–[9]. A recent population-based study carried out among adult patients who were treated in several Spanish primary care health centres revealed five multimorbidity patterns (cardiometabolic, mechanical, psychogeriatric, psychiatric-substance abuse, and depressive) [10]. This paper describes an external validation of these results, by applying the same methodology to data from another geographical setting with comparable socio-economic and health care features.

Further knowledge regarding the similarities and differences between the patterns obtained in different countries is expected to pave the way for the establishment of a sound and rigorous methodology for the identification of patterns of multimorbidity.

The present work aims to assess the similarities among the multimorbidity patterns identified in primary care patients from two European regions (Spain and the Netherlands) with similar organisational features of their primary care systems, using validated methods.

## Methods

### Study population and variables

Data for the Spanish population were obtained from electronic medical records of people over 14 years of age who consulted their general practitioner at least once in 2008 at any of the 19 primary health care centres included in this study. The centres were located in two north-eastern regions of Spain and had more than two years of experience using electronic records by all general practitioners and nurses [10]. Each primary care health centre has a geographically delimited population assigned to it and all types of patients and health problems, except for medical emergencies, are initially attended there.

Data for the Dutch population derived from the Registration Network Family Practices of The Netherlands (RegistratieNet Huisartspraktijken, RNH) [11]. The RNH is a dynamic and computerised database starting in 1987 which contains the medical records of 21 family practices in the south of the Netherlands, currently covering information of over 80,000 patients. General practitioners taking part in the RNH continuously register patient characteristics and relevant health problems and use of medication according to standardised procedures. The quality of the data is ascertained by ample instructions and training sessions, regular regional consensus groups, quality control audits, an online thesaurus available during data-entry and systematic control for erroneous or missing entries.

Both primary care databases contain basic demographics as well as all relevant health problems affecting the present functional status of patients and/or their future functioning coded using the International Classification of Primary Care (ICPC) [12]. To guarantee the comparability and quality of the data, only the episodes of those patients who remained in the database for the entire study years (2008 and 2010, respectively) were included in the study.

To facilitate the management of diagnostic information, diseases were grouped according to the Expanded Diagnostic Clusters (EDC) of the ACG system in both contexts. This system groups ICPC codes into 260 EDCs based on the clinical, diagnostic and therapeutic similarities of diseases [13]. The selection of chronic EDCs was similar to a previously validated list containing 114 EDCs [14].

All information was anonymised using a unique patient identifier. The Spanish database was approved by the Clinical Research Ethics Committee of Aragon (CEICA). In the Netherlands, no ethical approval is necessary when analysing anonymised data [15].

### Statistical analysis

The statistical analysis applied to the Dutch data was the same as that performed in the original Spanish study, and previously by other authors [16]. More specifically, an exploratory factor analysis was conducted analysing the clustering of the 114 selected EDCs. This statistical technique allows for groups of correlated diseases (i.e. factors) to be identified, under the assumption that they share some underlying common trait. In addition, it enables that a given disease belongs to more than one factor at the same time. The factors resulting from this analysis were interpreted as multimorbidity patterns (i.e. diseases non-randomly associated to each other). Given that exploratory factor analysis requires data to be in a continuous format, which is not feasible for diagnostic variables of a dichotomous nature, we employed tetra-choric correlation matrices where no assumptions are made on the distribution of the variables [17].

The extraction of the disease patterns was based on the principal factor method, and the number of factors to extract was established using a scree plot [18]. The scree plots obtained in both contexts can be seen in the supplemental file (File S1). An oblique rotation (Oblimin) was performed to allow a better interpretation of the factor analysis. The sampling adequacy was analysed using the Kaiser-Meyer-Olkin (KMO) parameter, and the cumulative fraction of total variance was used as a measure of the model's goodness of fit.

To determine which health problems (EDCs) belonged to each of the multimorbidity patterns, those with scores equal to or greater than 0.25 for each factor were selected. To calculate the prevalence of the patterns according to the most common definition of multimorbidity, an individual was assigned a specific pattern of multimorbidity if he/she presented at least two of the diseases included in the pattern. The entire analysis was stratified by age groups (15–44, 45–64, ≥65) and sex.

As in the original work in the Spanish setting, only diagnoses with a prevalence equal to or greater than 1% in each age and sex group were included in the analyses so as to increase the epidemiological interest of the study.

The STATA 12.0 software was used to conduct the statistical analysis.

## Results

The Dutch study population consisted of 79,291 individuals and the Spanish one of 275,682, with a slightly smaller proportion of middle age individuals in the latter group (Table 1). Multimorbidity was present in all age and sex groups, and increasing with age in both study populations. However, for all age and sex groups in the Dutch setting the number of chronic conditions with a prevalence equal to or greater than 1% was higher compared to the Spanish population, resulting in a higher prevalence of multimorbidity (43.8% vs. 36.8%). Also, a higher number of diseases per multimorbidity pattern was found in the Netherlands in comparison to Spain (an average of 12 vs. six).

**Table 1:** Description of study populations.

	Males					Females				Total
	15–44	45–64		≥65	Total	15–44	45–64	≥65	Total	
Distribution of the population										
NL	15,600 (40.67%)	14,823 (38.6%)		7,959 (20.7%)	38,382	16,045 (39.2%)	14,882 (36.4%)	9,982 (24.4%)	40,909	79,291
SP	54,705 (45.7%)	35,587 (29.6%)		29,361 (24.5%)	119,653	66,540 (42.6%)	46,035 (29.5%)	43,454 (27.8%)	156,029	275,682
Number of total chronic diseases (EDC)										
NL	74	78		78	—	74	78	76	—	82
SP	76	79		79	—	76	78	77	—	84
Number of chronic diseases (EDC) with prevalence ≥1%										
NL	20	41		54	—	21	44	52	—	62
SP	13	30		38	—	14	23	34	—	46
Prevalence of multimorbidity (%)										
NL	17.4	46.8		80.5	—	21.4	48.6	80.4	—	43.8
SP	11.0	39.2		64.8	—	16.2	46.9	69.3	—	36.8
Number of patterns of multimorbidity										
NL	3	2		2	—	3	2	3	—	—
SP	2	2		3	—	2	3	4	—	—

NL: The Netherlands; SP: Spain; EDC: Expanded Diagnostic Cluster.

### Multimorbidity patterns in Spain

In the original Spanish study [10] five multimorbidity patterns were identified: cardiometabolic, mechanical, psychiatric-substance abuse, depressive, psychogeriatric (Table 2). The first two showed a progression over time with the gradual inclusion of expected disease complications. Three of them affected both sexes (cardiometabolic, mechanical and psychogeriatric), and two of them were present exclusively in men or women (psychiatric-substance abuse and depressive, respectively) (Tables 3, 4, 5, 6, 7, and 8).

**Table 2:** Diseases included in the multimorbidity patterns identified in the Spanish population.

Pattern	Diseases
**Cardiometabolic**	Acute myocardial infarction, Cardiac arrhythmia, Cardiovascular disorders (other), Chronic liver disease, Congestive heart failure, Diabetes, Disorders of lipoid metabolism, Emphysema/chronic bronchitis/Chronic Obstructive Pulmonary Disease (COPD), Generalized atherosclerosis, Gout, Haematologic disorders (other), Hypertension, Iron deficiency/other deficiency anaemias, Ischemic heart disease, Obesity and Substance use.
**Mechanical**	Anxiety/neuroses, Arthropathy, Behaviour problems, Cervical pain syndromes, Deafness/hearing loss, Dermatitis/eczema, Disease of hair and hair follicles, Disorders of lipoid metabolism, Diverticular disease of colon, Gastro-oesophageal reflux (GERD), Iron deficiency/other deficiency anaemia, Low back pain, Obesity, Osteoporosis, Prostatic hypertrophy, Thyroid disease, Varicose veins of lower extremities.
**Psychiatric-substance abuse**	Affective psychosis, Anxiety/neuroses, Arthropathy, Behaviour problems, Cerebrovascular disease, Cervical pain syndromes, Chronic ulcer of the skin, Congestive heart failure, Dementia/delirium, Dermatitis/eczema, Depression, Disease of hair and hair follicles, Disorders of lipoid metabolism, Diverticular disease of colon, GERD, Iron deficiency/other deficiency anaemia, Low back pain, Neuroses, Obesity, Osteoporosis, Parkinson's disease, Prostatic hypertrophy, Schizophrenia/affective psychosis, Substance use, Thyroid disease and Varicose veins of lower extremities.
**Depressive**	Behaviour problems and Depression.
**Psychogeriatric**	Behaviour problems, Cardiac arrhythmia, Cerebrovascular disease, Chronic ulcers of the skin, Congestive heart failure, Dementia/delirium, Iron deficiency/other deficiency anaemias, Osteoporosis and Parkinson's disease.

**Table 3:** Patterns in males 15 to 44 years of age.

	Prevalence of diseases (%)		NL*			SP	
	NL	SP	Factor 1	Factor 2	Factor 3	Cardiometabolic	Psychiatric-substance abuse
Prevalence of patterns (%)	---	---	1.17	4.37	1.35	0.93	1.53
**Allergy**							
Asthma	8.27	2.28	0.08	0.09	−0.22	−0.1	0.04
**Cardiovascular**							
Disorders of lipid metabolism	1.58	4.54	**0.88**	0.00	−0.01	**0.42**	**0.29**
Hypertension	1.69	1.83	**0.58**	−0.1	**0.29**	**0.65**	0.13
**Ear, Nose, Throat**							
Deafness, hearing loss	1.24	0.47	−0.01	0.03	0.09		
**Endocrine**							
Diabetes	1.1	1.09	**0.88**	0.00	−0.11	**0.46**	0.05
Thyroid disease	0.21	1.24				0.14	0.15
**Gastrointestinal/Hepatic**							
Gastro-oesophageal reflux	1.18	0.83	0.11	**0.32**	**0.39**		
**Musculoskeletal**							
Arthropathy	3.55	4.47	0.08	0.08	**0.49**	0.12	−0.03
Cervical pain syndromes	0.67	2.39				0.14	0.02
Kyphoscoliosis	1.13	0.28	−0.79	0.17	**0.44**		
Low back pain	4.11	9.02	0.05	0.06	**0.64**	0.04	−0.02
**Neurologic**							
Developmental disorder	3.57	0.1	−0.16	**0.55**	−0.46		
Seizure disorder	1.1	0.52	−0.05	**0.32**	−0.04		
**Nutrition**							
Obesity	3.06	1.75	**0.52**	0.13	0.2	**0.45**	**0.26**
**Psychosocial**							
Anxiety, neuroses	6.26	6.69	0.00	**0.74**	0.14	0.03	**0.39**
Attention deficit disorder	1.67	0.17	−0.15	**0.63**	−0.41		
Behaviour problems	2.02	0.99	0.04	**0.3**	−0.02		
Depression	2.26	0.43	0.08	**0.59**	**0.44**		
Personality disorders	1.88	0.39	0.03	**0.59**	0.15		
Schizophrenia and affective psychosis	0.85	1.01				0.01	**0.36**
Substance use	3.19	1.51	0.03	**0.53**	0.11	−0.08	**0.53**
**Skin**							
Dermatitis and eczema	6.7	4.08	0.02	0.04	−0.07	0.09	0.03
Psoriasis	1.59	0.67	0.06	0.04	0.16		

The scores of those health problems belonging to each factor are highlighted in bold.

NL: The Netherlands; SP: Spain.

*KMO: 0.61.

*Percentage of cumulative variance: 37.78%.

**Table 4:** Patterns in males 45 to 64 years of age.

	Prevalence (%)		NL*		SP	
	NL	SP	Factor 1	Factor 2	Cardiometabolic	Mechanical
Prevalence of patterns (%)	—	—	19.41	11.36	9.20	4.86
**Allergy**						
Asthma	5.36	1.31	−0.01	0.19	−0.08	0.23
**Cardiovascular**						
Acute myocardial infarction	1.81	1.75	**0.52**	−0.13	**0.3**	−0.03
Cardiac arrhythmia	2.66	1.42	**0.42**	−0.05	**0.44**	−0.08
Cardiac valve disorders	1.33	0.36	**0.42**	−0.06		
Cardiovascular disorders, other	2.73	1.07	**0.39**	0.10	0.19	0.02
Disorders of lipid metabolism	11.1	19.25	**0.53**	0.02	0.24	0.17
Generalised atherosclerosis	2.35	1.19	**0.47**	0.09	**0.45**	−0.01
Hypertension	17.1	20.8	**0.57**	0.00	**0.42**	0.14
Ischemic heart disease (excluding AMI)	5.73	1.86	**0.63**	0.01	**0.35**	−0.08
**Ear, Nose, Throat**						
Deafness, hearing loss	4.47	1.13	0.04	0.17	−0.08	**0.26**
**Endocrine**						
Diabetes	8.37	10.22	**0.69**	−0.04	**0.46**	−0.07
Other endocrine disorders	1.65	0.69	**0.26**	0.17		
Thyroid disease	0.92	2.33			0.19	0.06
**Eye**						
Cataract, aphakia	1.44	0.89	**0.32**	−0.01		
Glaucoma	0.66	1.37			0.10	0.10
Retinal disorders (excluding diabetic retinopathy)	1.65	0.48	**0.65**	−0.28		
**Gastrointestinal/Hepatic**						
Chronic liver disease	1.17	1.49	0.16	**0.41**	**0.33**	−0.06
Diverticular disease of colon	1.3	0.29	0.12	**0.27**		
Gastro-oesophageal reflux	3.83	1.8	0.03	**0.31**	−0.05	**0.34**
Inflammatory bowel disease	1.05	0.24	−0.06	0.16		
Irritable bowel syndrome	1.43	0.37	−0.02	**0.37**		
**General Surgery**						
Varicose veins of lower extremities	1.78	1.42	0.1	0.05	0.01	**0.29**
**Genito-urinary**						
Prostatic hypertrophy	1.98	4.83	0.16	0.13	0.08	**0.30**
Renal calculi	2.79	1.55	0.12	0.04	0.06	0.15
**Hematologic**						
Hematologic disorders, other	0.6	1.52			0.21	0.02
**Malignancies**						
Malignant neoplasms of the skin	1.52	0.05	0.06	0.07		
**Musculoskeletal**						
Arthropathy	11.67	9.32	0.14	0.21	0.02	**0.25**
Cervical pain syndromes	3.54	3.12	0.04	**0.32**	−0.01	0.22
Kyphoscoliosis	1.98	0.08	−0.02	0.15		
Low back pain	14.63	13.02	0.05	**0.31**	−0.02	**0.28**
**Neurologic**						
Cerebrovascular disease	2.48	0.96	**0.41**	0.06		
Neurologic disorders, other	1.52	0.38	0.09	**0.29**		
Peripheral neuropathy, neuritis	3.67	1.03	**0.32**	0.22	0.09	0.12
Seizure disorder	1.34	0.51	0.06	0.22		
**Nutrition**						
Obesity	8.42	3.41	**0.51**	0.11	**0.27**	**0.26**
**Psychosocial**						
Anxiety, neuroses	6.69	8.85	−0.03	**0.48**	0.17	0.1
Behaviour problems	2.29	0.9	0.12	**0.36**		
Depression	4.74	0.86	−0.08	**0.51**		
Personality disorders	2.02	0.22	−0.27	**0.69**		
Schizophrenia and affective psyc.	0.64	1.01			0.02	−0.08
Substance use	4.39	1.98	−0.02	**0.56**	**0.34**	−0.14
**Respiratory**						
Emphysema, chronic bronchitis, COPD	4.35	2.34	0.11	**0.29**	**0.36**	0.09
**Rheumatologic**						
Gout	3.42	1.39	**0.35**	0.09	0.16	0.17
**Skin**						
Dermatitis and eczema	5.39	5.14	0.04	0.12	0.05	0.23
Psoriasis	3.61	1.13	0.06	0.04	0.04	0.06

The scores of those health problems belonging to each factor are highlighted in bold.

NL: The Netherlands; SP: Spain.

*KMO: 0.68.

*Percentage of cumulative variance: 18.98%.

**Table 5:** Patterns in males 65 years of age and older.

	Prevalence (%)		NL*		SP		
	NL	SP	Factor 1	Factor 2	Cardiometabolic	Psycho-geriatric	Mechanical
Prevalence of patterns (%)	—	—	66.55	2.99	21.20	2.36	13.56
**Allergy**							
Asthma	5.21	1.27	0.22	0.12	−0.12	−0.08	0.18
**Cardiovascular**							
Acute myocardial infarction	5.3	3.21	**0.27**	0.02	0.23	−0.02	−0.06
Cardiac arrhythmia	11.01	5.88	**0.48**	−0.11	**0.34**	0.19	0.09
Cardiac valve disorders	5.28	0.76	**0.46**	−0.08			
Cardiovascular disorders, other	8.49	2.55	**0.43**	−0.09	**0.25**	0.05	0.00
Congestive heart failure	4.05	1.83	**0.62**	−0.09	**0.52**	**0.25**	0.03
Disorders of lipid metabolism	17.78	17.69	**0.29**	0.18	0.09	−0.17	0.17
Generalised atherosclerosis	9.45	3.28	**0.49**	0.01	**0.28**	0.17	0.08
Hypertension	35.16	40.95	**0.32**	0.05	**0.34**	−0.10	0.09
Ischemic heart disease (excluding AMI)	21.62	5.52	**0.52**	0.01	0.20	0.04	0.18
**Ear, Nose, Throat**							
Deafness, hearing loss	15.78	1.77	**0.36**	−0.07	−0.04	−0.11	0.14
**Endocrine**							
Diabetes	19.27	19.72	**0.41**	0.11	**0.36**	0.03	−0.09
Osteoporosis	2.44	1.18	0.2	−0.18	−0.21	**0.35**	**0.40**
Other endocrine disorders	2.81	0.75	**0.29**	0.20			
Thyroid disease	1.73	2.81	**0.27**	−0.05	0.06	0.16	0.20
**Eye**							
Cataract, aphakia	12.44	6.03	**0.47**	−0.13	0.09	−0.05	0.19
Glaucoma	2.84	3.49	0.10	−0.27	0.04	0.00	0.11
Retinal disorders (excluding diab. retin.)	5.43	0.82	**0.33**	−0.06			
**Gastrointestinal/Hepatic**							
Chronic liver disease	1.23	0.96	0.10	**0.53**			
Diverticular disease of colon	5.48	0.77	**0.27**	0.17			
Gastro-oesophageal reflux	5.91	2.4	0.23	0.14	−0.06	0.09	**0.30**
Inflammatory bowel disease	1.27	0.2	0.20	0.01			
Irritable bowel syndrome	1.61	0.3	−0.08	**0.49**			
**General Surgery**							
Varicose veins of lower extremities	3.33	2.24	0.18	−0.02	0.05	0.05	0.19
**Genito-urinary**							
Prostatic hypertrophy	11.35	15.08	0.14	0.02	−0.07	−0.04	**0.31**
Prostatitis	1.31	0.66	−0.13	0.02			
Renal calculi	4.01	1.03	0.19	−0.07			
**Hematologic**							
Hematologic disorders, other	1.38	2.64	0.23	**0.33**	**0.35**	−0.07	0.01
Iron deficiency, other deficiency anaemias	2.75	4.47	**0.46**	−0.08	**0.29**	**0.33**	0.15
Thrombophlebitis	1.33	0.78	0.03	−0.10			
**Malignancies**							
Mild malignant neoplasms	1.8	2.42	0.05	**0.32**	0.00	0.05	0.14
Malignant neoplasms of the skin	5.84	0.21	0.17	−0.01			
Malignant neoplasms, bladder	1.73	0.81	0.07	−0.19			
Malignant neoplasms, colorectal	2.59	1.42	0.16	−0.19	0.07	0.07	0.00
Malignant neoplasms, prostate	5.64	3.06	0.11	−0.17	−0.03	0.19	−0.02
**Musculoskeletal**							
Arthropathy	19.78	13.65	**0.32**	0.1	0.03	−0.02	**0.29**
Cervical pain syndromes	5.54	2.79	0.18	0.15	−0.03	−0.06	**0.29**
Kyphoscoliosis	2.53	0.08	0.11	−0.02			
Low back pain	20.49	13.44	**0.26**	0.07	−0.01	−0.02	**0.36**
**Neurologic**							
Cerebrovascular disease	10.29	3.78	**0.40**	−0.20	0.20	**0.25**	0.00
Dementia and delirium	2.64	4.04	0.23	−0.48	−0.08	**0.38**	0.10
Neurologic disorders, other	2.02	0.48	0.18	0.00			
Parkinson's disease	1.32	1.25	0.05	−0.64	0.02	**0.35**	0.00
Peripheral neuropathy, neuritis	6.22	1.37	**0.34**	0.16	0.19	0.01	0.15
Seizure disorder	1.38	0.52	0.11	−0.47			
**Nutrition**							
Obesity	9.47	3.12	**0.38**	**0.27**	**0.29**	−0.22	0.04
**Psychosocial**							
Anxiety, neuroses	5.82	6.63	0.13	0.18	0.03	0.20	**0.28**
Behaviour problems	1.31	1.19	0.2	**0.42**	0.04	**0.28**	0.04
Depression	3.24	0.74	0.18	−0.04			
Substance use	3.02	0.83	0.11	**0.25**			
**Reconstructive**							
Chronic ulcer of the skin	0.54	1.14			0.23	**0.59**	−0.15
**Respiratory**							
Emphysema, chronic bronchitis, COPD	13.03	9.57	**0.33**	0.00	0.15	0.12	0.24
**Rheumatologic**							
Gout	6.45	2.02	**0.32**	0.21	**0.27**	−0.08	0.03
**Skin**							
Dermatitis and eczema	5.73	7.76	0.15	−0.03	0.00	0.04	**0.32**
Psoriasis	4.23	1.06	0.12	**0.42**	0.04	−0.08	0.11

The scores of those health problems belonging to each factor are highlighted in bold.

NL: The Netherlands; SP: Spain.

*KMO: 0.66.

*Percentage of cumulative variance: 13.17%.

**Table 6:** Patterns in females 15 to 44 years of age.

	Prevalence (%)		NL*				SP	
	NL	SP		Factor 1	Factor 2	Factor 3	Cardiometabolic	Mechanical
Prevalence of patterns (%)	—	—		8.30	4.26	0.17	0.38	3.69
**Allergy**								
Asthma	8.41	2.39		**0.35**	−0.02	0.15	0.18	−0.02
**Cardiovascular**								
Disorders of lipid metabolism	0.91	2.21					**0.33**	0.16
Hypertension	1.87	1.06		**0.34**	0.15	−0.09	**0.60**	0.04
**Endocrine**								
Other endocrine disorders	2.4	0.94		**0.31**	0.01	0.02		
Thyroid disease	1.31	4.33		**0.53**	−0.09	−0.04	0.14	**0.25**
**Gastrointestinal/Hepatic**								
Irritable bowel syndrome	3.17	0.32		0.20	**0.27**	−0.03		
**General Surgery**								
Varicose veins of lower extremities	2.08	1.75		**0.27**	−0.13	−0.59	−0.02	**0.36**
**Hematologic**								
Iron deficiency, other deficiency anemias	0.75	4.38					0.03	**0.30**
**Musculoskeletal**								
Arthropathy	3.89	4.82		**0.58**	0.02	0.04	0.09	**0.26**
Cervical pain syndromes	1.26	4.43		0.08	0.22	−0.84	−0.04	**0.32**
Kyphoscoliosis	1.62	0.41		**0.29**	−0.24	**0.58**		
Low back pain	4.33	10.35		**0.47**	0.17	−0.20	0.05	0.22
**Neurologic**								
Developmental disorder	1.87	0.11		−0.02	0.22	**0.89**		
Peripheral neuropathy, neuritis	1.57	0.91		**0.57**	0.12	−0.05		
Seizure disorder	1.04	0.44		0.03	0.24	**0.31**		
**Nutrition**								
Obesity	4.77	2.55		**0.56**	0.10	0.01	**0.56**	0.09
**Psychosocial**								
Anxiety, neuroses	8.08	11.69		0.03	**0.73**	0.09	0.10	0.22
Behaviour problems	1.86	1.75		0.09	**0.49**	0.04	0.04	**0.27**
Depression	4.67	0.86		0.13	**0.57**	−0.10		
Personality disorders	2.64	0.32		−0.07	**0.89**	−0.01		
Substance use	1.27	0.29		−0.14	**0.69**	0.04		
**Skin**								
Dermatitis and eczema	9.51	4.75		**0.29**	−0.05	0.05	0.03	**0.29**
Disease of hair and hair follicles	0.47	1.28					−0.09	**0.26**
Psoriasis	1.7	0.47		**0.25**	−0.01	−0.11		

The scores of those health problems belonging to each factor are highlighted in bold.

NL: The Netherlands; SP: Spain.

*KMO: 0.61.

*Percentage of cumulative variance: 36.74%.

**Table 7:** Patterns in females 45 to 64 years of age.

	Prevalence (%)		NL*			SP			
	NL	SP		Factor 1	Factor 2		Cardiometabolic	Mechanical	Depressive
Prevalence of patterns (%)	—	—		21.26	8.02		4.04	16.64	0.12
**Allergy**									
Asthma	6.63	2.47		0.09	0.21		0.03	0.18	−0.01
**Cardiovascular**									
Cardiac arrhythmia	2.09	0.75		**0.44**	0.05				
Cardiac valve disorders	1.2	0.22		**0.36**	0.06				
Cardiovascular disorders, other	1.76	1.34		**0.54**	−0.04		0.07	0.17	0.02
Disorders of lipoid metabolism	7.84	16.22		**0.44**	0.02		0.21	0.19	−0.08
Generalised atherosclerosis	1.96	0.45		**0.33**	0.23				
Hypertension	15.98	15.72		**0.55**	−0.1		**0.77**	−0.11	0.02
Ischemic heart disease (excluding AMI)	2.14	0.4		**0.51**	0.14				
**Ear, Nose, Throat**									
Deafness, hearing loss	2.67	0.89		0.16	0.17				
**Endocrine**									
Diabetes	5.59	5.16		**0.61**	−0.06		**0.58**	−0.1	0.02
Osteoporosis	4.35	8.89		0.23	0.2		−0.03	**0.25**	−0.14
Other endocrine disorders	4.71	1.73		**0.26**	0.02		0.05	0.03	0.21
Thyroid disease	4.37	9.26		**0.3**	−0.04		0.07	**0.29**	−0.16
**Eye**									
Cataract, aphakia	1.42	0.86		**0.53**	−0.21				
Glaucoma	0.74	1.62					0.16	0.12	0.11
Retinal disorders (excluding diab. retin.)	1.18	0.37		**0.6**	−0.23				
**Female Reproductive**									
Utero-vaginal prolapse	2.18	0.56		0.2	−0.06				
**Gastrointestinal/Hepatic**									
Diverticular disease of colon	1.36	0.33		**0.34**	0.2				
Gastro-oesophageal reflux	3.32	2.1		**0.25**	**0.31**		0.05	**0.4**	0.04
Inflammatory bowel disease	1.24	0.19		−0.06	0.19				
Irritable bowel syndrome	3.25	0.57		0.06	**0.43**				
**General Surgery**									
Chronic cystic disease of the breast	2.51	0.73		0.05	0.08				
Renal calculi	1.2	0.93		0.14	0.1				
Varicose veins of lower extremities	7.22	4.93		0.18	0.07		0.01	**0.26**	0.07
**Hematologic**									
Hematologic disorders, other	0.94	1.31					0.08	0.13	−0.06
Iron deficiency, other deficiency anemias	1.59	4.26		0.13	0.21		−0.02	0.17	−0.02
**Malignancies**									
Mild malignant neoplasms	0.87	1.88					0	0.12	−0.33
Malignant neoplasms of the skin	2.3	0.05		0.07	−0.02				
Malignant neoplasms of the breast	2.92	0.28		0.02	−0.05				
**Musculoskeletal**									
Arthropathy	12.67	14.81		**0.28**	0.15		0.09	0.21	0.19
Cervical pain syndromes	4.02	6.15		0.08	**0.32**		−0.07	**0.29**	0.1
Kyphoscoliosis	1.88	0.3		0.06	0.18				
Low back pain	12.77	17.19		0.21	0.23		0.02	**0.28**	0.16
**Neurologic**									
Cerebrovascular disease	2.03	0.46		**0.42**	0.09				
Neurologic disorders, other	1.68	0.61		0.21	**0.33**				
Peripheral neuropathy, neuritis	4.91	2.46		**0.29**	0.15		0.03	0.23	0.06
Seizure disorder	1.05	0.4		−0.02	**0.34**				
**Nutrition**									
Obesity	8.48	5.49		**0.57**	0.02		**0.37**	0.16	0.03
**Psychosocial**									
Anxiety, neuroses	8.91	18.53		−0.05	**0.65**		−0.01	**0.34**	−0.16
Behaviour problems	1.46	1.8		0.02	**0.54**		−0.02	0.2	**0.29**
Depression	6.54	2.18		−0.08	**0.51**		−0.01	0.06	**0.68**
Personality disorders	1.99	0.32		−0.26	**0.77**				
Substance use	1.9	0.42		−0.12	**0.62**				
**Respiratory**									
Emphysema, chronic bronchitis, COPD	4.31	0.78		0.14	**0.48**				
**Skin**									
Dermatitis and eczema	7.96	5.47		0.08	0.11		0.02	**0.25**	0.05
Disease of hair and hair follicles	1.01	0.96		0.11	0.01				
Psoriasis	3.39	0.81		0.09	0.07				

The scores of those health problems belonging to each factor are highlighted in bold.

NL: The Netherlands; SP: Spain.

*KMO: 0.67.

*Percentage of cumulative variance: 17.59%.

**Table 8:** Patterns in females 65 years of age and older.

	Prevalence (%)		NL*				SP			
	NL	SP	Factor 1	Factor 2	Factor 3	Cardiometabolic		Psycho-geriatric	Mechanical	Depressive
Prevalence of patterns (%)	—	—	46.07	1.04	43.07	17.30		3.48	33.30	0.16
**Allergy**										
Asthma	5.38	2.87	−0.05	0.19	**0.31**	0.12		−0.02	0.23	−0.01
**Cardiovascular**										
Acute myocardial infarction	2.37	0.93	**0.46**	−0.01	−0.09					
Cardiac arrhythmia	9.89	5.04	**0.54**	−0.06	−0.03	**0.36**		**0.34**	−0.02	−0.14
Cardiac valve disorders	5.48	0.95	**0.53**	−0.11	−0.02					
Cardiovascular disorders, other	5.73	2.93	**0.51**	−0.05	0.1	0.21		0.1	0.07	0.09
Congestive heart failure	4.05	2.34	**0.74**	−0.04	−0.2	**0.37**		**0.39**	−0.04	0.03
Disorders of lipoid metabolism	18.99	20.31	0.24	0.17	0.13	0.04		−0.16	**0.25**	−0.06
Generalised atherosclerosis	6.36	1.24	**0.49**	0.09	0.02	0.09		0.23	0.07	−0.14
Hypertension	40.89	45.86	**0.44**	0.04	−0.02	**0.44**		−0.08	0.11	0.03
Ischemic heart disease (excluding AMI)	11.94	2.78	**0.56**	0.13	0.08	**0.29**		0.18	0.09	−0.06
**Ear, Nose, Throat**										
Deafness, hearing loss	11.54	1.77	**0.28**	−0.01	0.15	0.02		0.03	0.17	0.13
**Endocrine**										
Diabetes	17.82	14.83	**0.43**	−0.06	0.11	**0.46**		0.04	−0.13	−0.02
Osteoporosis	16.2	13.26	0.05	0.01	**0.25**	−0.17		−0.07	**0.4**	−0.07
Other endocrine disorders	7	1.49	**0.25**	0.14	0.15	0.14		0	0.1	0.18
Thyroid disease	6.4	7.22	0.17	0.1	0.11	0.02		0.01	**0.25**	−0.13
**Eye**										
Cataract, aphakia	16.49	7.28	**0.38**	−0.01	0.15	0.06		−0.01	0.22	0.09
Glaucoma	3.19	4.07	0.2	0.04	−0.01	0.07		−0.01	0.2	0.02
Retinal disorders (excluding diab. retin.)	6.91	0.86	**0.39**	0	0.12					
**Female Reproductive**										
Utero-vaginal prolapse	5.37	0.88	0.01	−0.16	**0.26**					
**Gastrointestinal/Hepatic**										
Chronic liver disease	1.03	0.79	0.07	0.07	**0.28**					
Diverticular disease of colon	6.9	1.23	0.15	−0.02	**0.34**	−0.08		0.14	**0.31**	0.01
Gastro-oesophageal reflux	6.95	3.37	0.08	−0.06	**0.39**	−0.14		0.13	**0.44**	0.09
Inflammatory bowel disease	1.09	0.13	0	**0.26**	−0.02					
Irritable bowel syndrome	3.13	0.66	−0.11	0.09	**0.45**					
**General Surgery**										
Chronic cystic disease of the breast	1.55	0.26	−0.42	−0.07	**0.43**					
Renal calculi	1.79	0.61	−0.08	**0.38**	0.22					
Varicose veins of lower extremities	8.8	7.58	−0.01	−0.34	**0.33**	0.05		0.03	**0.3**	−0.02
**Hematologic**										
Hematologic disorders, other	1.21	2.14	0.24	0	0.19	**0.31**		0.12	0.01	0.05
Iron deficiency, other deficiency anemias	3.26	6.05	**0.45**	−0.14	0.08	0.14		**0.35**	0.1	0
Thrombophlebitis	1.43	1	0.03	−0.54	0.2					
**Malignancies**										
Mild malignant neoplasms	1.9	2.52	0.05	**0.41**	−0.07	−0.05		0.1	0.11	−0.33
Malignant neoplasms of the skin	4.95	0.08	0.11	−0.04	0.03					
Malignant neoplasms, breast	4.97	0.21	0.07	−0.04	0					
Malignant neoplasms, colorectal	2.37	0.77	0.13	0.07	−0.07					
**Musculoskeletal**										
Arthropathy	30.78	21.55	0.15	−0.16	**0.42**	0.09		−0.04	**0.3**	0
Cervical pain syndromes	5.77	3.82	−0.06	0.2	**0.46**	−0.02		−0.09	**0.28**	0.01
Kyphoscoliosis	2.72	0.31	−0.09	−0.2	**0.48**					
Low back pain	19.15	18.03	−0.02	−0.03	**0.57**	0.05		−0.06	**0.34**	0.07
**Neurologic**										
Cerebrovascular disease	8.14	2.67	**0.45**	0.02	0.03	0.08		**0.36**	−0.03	0.08
Dementia and delirium	3.58	6.41	**0.36**	0.01	−0.15	−0.13		**0.42**	−0.01	0
Neurologic disorders, other	2.39	0.55	0.02	0.05	**0.41**					
Peripheral neuropathy, neuritis	7.29	1.83	0.21	−0.06	**0.37**	0.1		−0.05	0.15	−0.17
**Nutrition**										
Obesity	11.16	4.96	**0.29**	−0.1	**0.27**	**0.3**		−0.25	0.13	0.13
**Psychosocial**										
Anxiety, neuroses	8.62	15.18	0	0.24	**0.35**	−0.07		0.18	**0.37**	−0.13
Behaviour problems	1.65	2.02	0.15	**0.42**	0.09	−0.06		0.19	0.17	**0.39**
Depression	5.49	1.97	0.05	**0.25**	0.22	0.05		−0.06	0.06	**0.79**
Substance use	1.77	0.12	−0.04	**0.37**	0.22					
**Reconstructive**										
Chronic ulcer of the skin	1.08	1.67	0.23	−0.78	0.03	0.1		**0.5**	−0.16	0.02
**Respiratory**										
Emphysema, chronic bronchitis, COPD	7.19	2.42	0.14	0.17	**0.26**	0.05		0.18	0.15	0
**Rheumatologic**										
Gout	2.23	0.32	**0.43**	0.01	−0.07					
**Skin**										
Dermatitis and eczema	5.88	6.67	−0.01	−0.21	**0.27**	0.03		0.02	**0.27**	0.04
Psoriasis	4.43	0.6	0.06	0.02	0.15					

The scores of those health problems belonging to each factor are highlighted in bold.

NL: The Netherlands; SP: Spain.

*KMO: 0.69.

*Percentage of cumulative variance: 19.02%.

### Multimorbidity patterns in the Netherlands

#### Young Dutch men

In total, 6.3% of young Dutch men were assigned to at least one of the three factors obtained for this age group (Table 3). Factor 1 clustered cardiovascular conditions, diabetes and obesity, which are also present in the Spanish cardiometabolic pattern. Factor 2 clustered diseases similar to the Spanish psychiatric-substance abuse pattern, given the common presence of psychosocial and neurologic diseases, and gastro-oesophageal reflux, but with additional psychosocial and neurologic diseases in the former case. Factor 3, finally, combined musculoskeletal conditions, gastro-oesophageal reflux, hypertension and depression.

#### Middle age Dutch men

Over a quarter of Dutch men in this age group (27.3%) were assigned to at least one of the two identified factors (Table 4). The first one was similar to Factor 1 found for young men, but also included cardiovascular, endocrine, neurologic and eye disorders, and gout. Many of the diseases found, especially the cardiovascular disorders, were also present in the Spanish cardiometabolic pattern. The second factor comprised psychosocial, gastrointestinal/hepatic, musculoskeletal and neurologic conditions, as well as COPD. Two of these diseases (gastro-oesophageal reflux and low back pain) were also present in the Spanish factor covering the mechanical pattern.

#### Older Dutch men

Two thirds of the men in this age group (66.9%) were assigned to at least one of the two factors identified for this group (Table 5). Factor 1 (with a prevalence as high as 66.6%), clustered a number of diseases, amongst which cardiovascular diseases, diabetes, thyroid disease, obesity and a variety of other disorders. This group of disorders showed manifest similarities to the Spanish cardiometabolic pattern, given that over one third of the diseases comprising the Dutch factor (various cardiovascular diseases, diabetes, iron deficiency, obesity and gout) was also present in the Spanish results. Factor 2 encompassed various psychosocial and gastrointestinal-hepatic diseases, hematologic disorders, mild malignancies, obesity and psoriasis.

#### Young Dutch women

At least one of the three factors identified for young Dutch women were present in 12% of this group (Table 6). Factor 1 included musculoskeletal diseases, obesity, thyroid disease and other endocrine and skin disorders along with other health problems. Some of these diseases (thyroid disease, varicose veins, arthropathy and dermatitis/eczema) were also included in the Spanish mechanical pattern. Factor 2 comprised psychosocial diseases and irritable bowel syndrome, and in Factor 3 neurologic diseases and kyphoscoliosis were associated.

#### Middle age Dutch women

Two patterns affecting more than a quarter of these women (26.2%) were identified (Table 7). Factor 1 covered cardiovascular diseases, diabetes, obesity, arthropathy, thyroid disease and other disorders. Three of these diseases (hypertension, diabetes and obesity) were also present in the Spanish cardiometabolic pattern. Factor 2 consisted of psychosocial, neurologic and gastrointestinal disorders, in addition to cervical pain and COPD. Again, three of these conditions (gastro-oesophageal reflux, cervical pain syndrome and anxiety/neuroses) coincided with the Spanish mechanical pattern, and two (behaviour problems and depression) with the Spanish depressive pattern.

#### Older Dutch women

Almost two out of three women (64.8%) were assigned to at least one of the three factors identified in this age group (Table 8). The first factor, affecting almost half of them (46.1%), was conformed by cardiovascular diseases, diabetes and other endocrine, eye and neurologic disorders, as well as obesity, gout, iron deficiency and deafness. This factor clearly reminded of the Spanish cardiometabolic pattern. Several other conditions of this first factor were included in the Spanish psychogeriatric pattern (congestive heart failure, cardiac arrhythmia, iron deficiency, cerebrovascular disease and dementia). Factor 2 included psychological diseases, also included in the Spanish depressive pattern, along with inflammatory bowel disease, renal calculi and mild malignancies. Finally, Factor 3 with a prevalence as high as 43.1%, grouped musculoskeletal and gastrointestinal/hepatic disorders, among others. Half of these diseases were also found in the Spanish mechanical pattern (osteoporosis, gastro-oesophageal reflux, diverticular disease of the colon, varicose veins, arthropathy, cervical pain, low back pain, anxiety/neuroses, and dermatitis/eczema).

## Discussion

### Main findings

In this first population-based study comparing multimorbidity patterns in two different European regions, relevant similarities were found for three of the five patterns identified in the original Spanish study, namely the cardiometabolic, mechanical and psychiatric-substance abuse. The analysis of Dutch data revealed a higher prevalence of multimorbidity which corresponds with the clustering of a higher number of diseases in each of the patterns.

### Interpretation of findings

To date, very few studies focusing on disease patterns encompass populations covering broad age ranges. This study attempts to take into account health problems throughout the entire lifespan, supporting the generalizability of the findings [19]. Additionally, previous studies on multimorbidity patterns have used a variety of methodologies with respect to sample size, participant age, recruitment strategy, data sources, number of baseline diseases and statistical procedures. This heterogeneity makes comparisons between studies difficult [7].

The pattern with highest similarities in the Spanish and Dutch results was the cardiometabolic one. This pattern, as shown in the Spanish study [10] and also recently brought to light in a systematic review [7], was present in all age and sex groups, except in young Dutch women. However, for the Dutch population the pattern presented earlier in life and with more complex health problems. For example, cardiovascular diseases such as acute myocardial infarction and cardiac arrhythmia, which appeared in the Dutch population at middle ages, were only present in Spanish women of older age.

The greater complexity of multimorbidity patterns in the Dutch population starting at middle age could be partly explained by the use of different diagnostic protocols in the two countries. In this regard, it is worth mentioning that in 2010 functional payments for the total episode of care were introduced in general practice in the Netherlands for specific conditions (diabetes, COPD, heart failure) and cardiovascular risk management, under the premise that this new payment system would improve the position of the patient and the quality of care [20]. It could be argued that this type of measures has enhanced the active detection and management of cardiovascular conditions.

In the Dutch population, a mechanical component was evident in young and elderly women and in men up to middle age, comparable to the Spanish mechanical pattern. Diseases such as arthropathy, cervical and low back pain, varicose veins of the lower extremities, gastro-oesophageal reflux, anxiety and obesity coincided in both studies and confirm previous results found by other authors [7], [21], [22]. However, in most of the Dutch age and sex groups, this pattern was expanded with psychosocial diseases (substance use, behavioural problems, personality disorders and depression). This was previously described in the literature, particularly for working-age populations [23], [24].

Two of the diseases associated with the psychiatric-substance abuse pattern described in young Spanish men (substance use and anxiety/neuroses) together with other psychosocial conditions were also present in the Dutch population, but in all age and sex groups. The recurrent presentation of this cluster of psychosocial problems in the Dutch population could be a consequence of the development in the past 15 years of the Dutch mental health sector; about half of Dutch adults with a serious mental or addictive disorder receive care and two-thirds of them receive satisfactory care [25].

### Strengths and limitations

The main strengths of the present study are the similarities concerning the study populations and the organisational features of primary care in both the Spanish and Dutch contexts. Both studies were based on primary care populations served by general practitioners who systematically register diagnoses using ICPC. Moreover, the public nature of both health care systems and the high access of citizens, as well as the one-year observation periods guarantee that selection bias is reduced. Still, several limitations need to be considered.

Diagnostic habits and registration of clinical data may vary in the two countries, due to differences in the level of professional training, the degree of implementation or content of clinical practice guidelines, the use of active protocols for detecting certain diseases, organisational factors, etc. Indeed, the Dutch general practice holds an outstanding position regarding quality assurance and guideline implementation with respect to other European countries [20]. Regarding differences in the registration procedure, we must bear in mind that the Dutch database was designed not only for clinical but also research purposes, using quality improvement procedures. This could explain why some diseases have a higher prevalence in the Netherlands compared to Spain. Nevertheless, essential methodological considerations for multimorbidity studies [26], such as the use of health information from primary care patients and the coding of morbidity was identical in both contexts.

Another limitation, also mentioned in the original Spanish study, was the cross-sectional design used in both studies, limiting the establishment of causal relationships and/or the evolution of patterns over time [27].

The study of the Dutch patterns from the perspective of the pathophysiology of the identified disease interactions was out of the scope of this study, focused rather on comparing the multimorbidity patterns obtained in two different health care contexts. As a consequence, Dutch patterns lacked specific clinical labels.

The assessment of the differences/similarities between the results obtained in both countries was largely qualitative and descriptive. A statistical analysis of such differences does not seem very relevant in this study since, given the large numbers, all differences are expected to be statistically significant. Therefore, a qualitative comparison seems to be more pertinent and better adapted to the clinical appraisal of the associations and interactions among diseases.

### Implications for health care delivery and clinical practice

The similarities found for certain multimorbidity patterns in primary care patients from two different European countries could offer initial clues for the elaboration of clinical practice guidelines, if further evidenced in other contexts. This has been repeatedly mentioned and urgently requested by the scientific community [28]. Understanding the way in which pathologies are associated with one another throughout the lives of individuals, as well as knowing how frequently these diseases appear, will bring about a better understanding of multimorbidity. Indeed, the current definition of multimorbidity based on simple counts of conditions (e.g., two or more) is unenlightening with respect to addressing the health problems of multimorbid patients [29]; [30].

This study also endorses the use of primary care electronic medical records for the epidemiologic characterization of multimorbidity. Moreover, electronic medical records would enable a longitudinal approach to the multimorbidity phenomenon.

## Supporting Information

File S1 **Combined file of supporting figures.** Figure A - Scree plots for the different age and sex groups in the Spanish setting. Figure B - Scree plots for the different age and sex groups in the Dutch setting.
(DOC)

## References

[1] Barnett K, Mercer SW, Norbury M, Watt G, Wyke S, et al (2012) Epidemiology of multimorbidity and implications for health care, research, and medical education: a cross-sectional study. Lancet 380: 37–43. 22579043 10.1016/S0140-6736(12)60240-2

[2] Fortin M, Bravo G, Hudon C, Lapointe L, Almirall J, et al (2006) Relationship between multimorbidity and health-related quality of life of patients in primary care. Qual Life Res 15: 83–91. 16411033 10.1007/s11136-005-8661-z

[3] Gijsen R, Hoeymans N, Schellevis FG, Ruwaard D, Satariano WA, et al (2001) Causes and consequences of comorbidity: a review. J Clin Epidemiol 54: 661–74. 11438406 10.1016/s0895-4356(00)00363-2

[4] Wolff J, Starfield B, Anderson G (2002) Prevalence, expenditures, and complications of multiple chronic conditions in the elderly. Arch Intern Med 162: 2269–76. 12418941 10.1001/archinte.162.20.2269

[5] Mangin D, Heath I, Jamoulle M (2012) Beyond diagnosis: rising to the multimorbidity challenge. BMJ 344: e3526. 22695898 10.1136/bmj.e3526

[6] NICE website. NICE should produce guidance on multiple morbidities. Available: http://www.nice.org.uk/newsroom/news/NICEShouldProduceGuidanceOnMultipleMorbidities.jsp. Accessed 2012 Apr 26.

[7] Prados-Torres A, Calderon-Larranaga A, Hancco-Saavedra J, Poblador-Plou B, van den Akker M (2014) Multimorbidity patterns: a systematic review. J Clin Epidemiol 67: 254–66. 24472295 10.1016/j.jclinepi.2013.09.021

[8] Garin N, Olaya B, Perales J, Moneta MV, Miret M, et al (2014) Multimorbidity patterns in a national representative sample of the Spanish adult population. PLoS One 9: e84794. 24465433 10.1371/journal.pone.0084794 PMC3896355

[9] Islam MM, Valderas JM, Yen L, Dawda P, Jowsey T, et al (2014) Multimorbidity and comorbidity of chronic diseases among the senior Australians: prevalence and patterns. PLoS One 9: e83783. 24421905 10.1371/journal.pone.0083783 PMC3885451

[10] Prados-Torres A, Poblador-Plou B, Calderon-Larranaga A, Gimeno-Feliu LA, Gonzalez-Rubio F, et al (2012) Multimorbidity Patterns in Primary Care: Interactions among Chronic Diseases Using Factor Analysis. PloS One 7: e32190. 22393389 10.1371/journal.pone.0032190 PMC3290548

[11] Metsemakers JF, Hoppener P, Knottnerus JA, Kocken RJ, Limonard CB (1992) Computerized health information in The Netherlands: a registration network of family practices. Br J Gen Pract 42: 102–6. 1493025 PMC1371993

[12] Lamberts H, Wood M (1987) ICPC: International Classification of Primary Care. Oxford: Oxford University Press. 201 p.

[13] The Johns Hopkins ACG System (2008) Reference Manual, version 8.2.

[14] Salisbury C, Johnson L, Purdy S, Valderas JM, Montgomery AA (2011) Epidemiology and impact of multimorbidity in primary care: a retrospective cohort study. Br J Gen Pract 61: e12–e21. 21401985 10.3399/bjgp11X548929 PMC3020068

[15] van den Dungen C, Hoeymans N, Boshuizen HC, van den Akker M, Biermans MC, et al (2011) The influence of population characteristics on variation in general practice based morbidity estimations. BMC Public Health 11: 887. 22111707 10.1186/1471-2458-11-887 PMC3280203

[16] Schafer I, von Leitner EC, Schon G, Koller D, Hansen H, et al (2010) Multimorbidity patterns in the elderly: a new approach of disease clustering identifies complex interrelations between chronic conditions. PLoS One 5: e15941. 21209965 10.1371/journal.pone.0015941 PMC3012106

[17] Kubinger KD (2003) On artificial results due to using factor analysis for dichotomous variables. Psycology Science 45: 106–10.

[18] Tabchnic B, Fidell L (2006) Using Multivariate Statistics. Boston: Allyin & Bacon.

[19] France EF, Wyke S, Gunn JM, Mair FS, McLean G, et al (2012) Multimorbidity in primary care: a systematic review of prospective cohort studies. Br J Gen Pract 62: e297–e307. 22520918 10.3399/bjgp12X636146 PMC3310037

[20] Schäfer W, Kroneman M, Boerma W, van den Berg M, Westert G, et al (2010) The Netherlands: Health system review. Health Systems in Transition 12(1): 1–229. 21132996

[21] Cornell J, Pugh J, Williams JW (2007) Multimorbidity Clusters: Clustering Binary Data From Multimorbidity Clusters: Clustering Binary Data From A Large Administrative Medical Database. Applied Multivariate Research 12(3): 163–82.

[22] van den Bussche H, Koller D, Kolonko T, Hansen H, Wegscheider K, et al (2011) Which chronic diseases and disease combinations are specific to multimorbidity in the elderly? Results of a claims data based cross-sectional study in Germany. BMC Public Health 11: 101. 21320345 10.1186/1471-2458-11-101 PMC3050745

[23] Kraatz S, Lang J, Kraus T, Munster E, Ochsmann E (2013) The incremental effect of psychosocial workplace factors on the development of neck and shoulder disorders: a systematic review of longitudinal studies. Int Arch Occup Environ Health 86: 375–95. 23549669 10.1007/s00420-013-0848-y

[24] Olaya-Contreras P, Styf J (2013) Biopsychosocial function analyses changes the assessment of the ability to work in patients on long-term sick-leave due to chronic musculoskeletal pain: The role of undiagnosed mental health comorbidity. Scand J Public Health 41: 247–55. 23361388 10.1177/1403494812473380

[25] Wang PS, Aguilar-Gaxiola S, Alonso J, Angermeyer MC, Borges G, et al (2007) Use of mental health services for anxiety, mood, and substance disorders in 17 countries in the WHO world mental health surveys. Lancet 370: 841–50. 17826169 10.1016/S0140-6736(07)61414-7 PMC2847360

[26] Stewart M, Fortin M, Britt HC, Harrison CM, Maddocks HL (2013) Comparisons of multi-morbidity in family practice—issues and biases. Fam Pract 30: 473–80. 23666805 10.1093/fampra/cmt012 PMC3722508

[27] Mercer SW, Gunn J, Wyke S (2011) Improving the health of people with multimorbidity: the need for prospective cohort studies. J Comorb 1: 4–7. 29090130 10.15256/joc.2011.1.10 PMC5556415

[28] Hughes LD, McMurdo ME, Guthrie B (2012) Guidelines for people not for diseases: the challenges of applying UK clinical guidelines to people with multimorbidity. Age Ageing 42(1): 62–9. 22910303 10.1093/ageing/afs100

[29] Caughey GE, Roughead EE (2011) Multimorbidity research challenges: where to go from here? J Comorb 1: 8–10. 29090131 10.15256/joc.2011.1.9 PMC5556416

[30] Goodman RA, Parekh AK, Koh HK (2012) Toward a more cogent approach to the challenges of multimorbidity. Ann Fam Med 10: 100–1. 22412000 10.1370/afm.1391 PMC3315138

