# Supplementary material for: Similar Multimorbidity Patterns in Primary Care Patients from Two European Regions: Results of a Factor Analysis
Source: PLoS One. 2014 Jun 23;9(6):e100375. doi: 10.1371/journal.pone.0100375 (PMC4067297; doi:10.1371/journal.pone.0100375)

Supplemental file (S1)

Figure A - Scree plots for the different age and sex groups in the Spanish setting


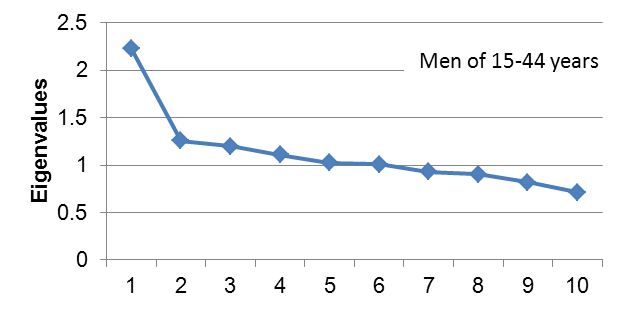

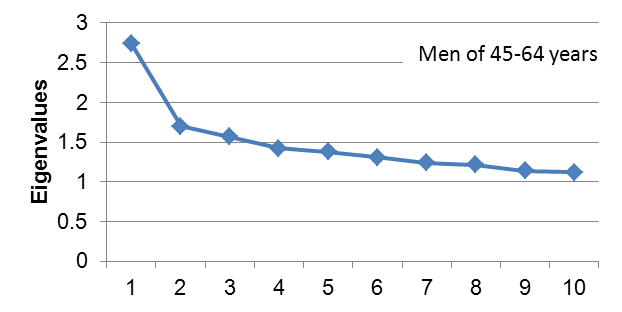

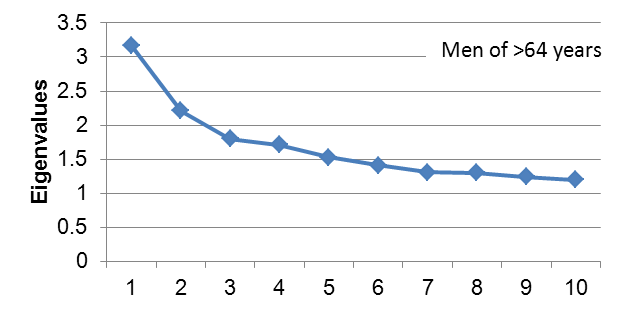


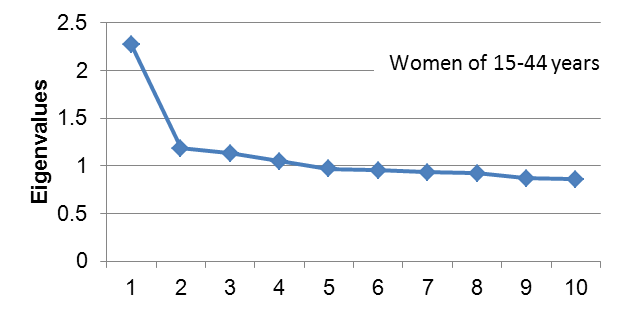

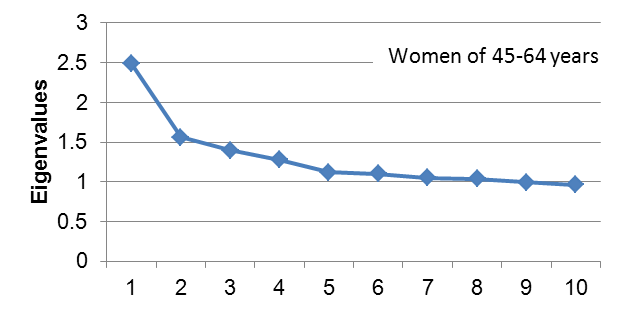

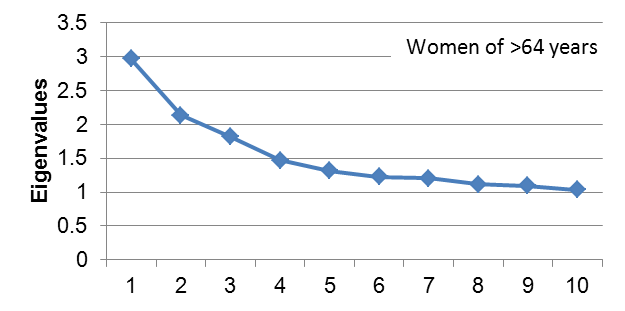


Figure B - Scree plots for the different age and sex groups in the Dutch setting


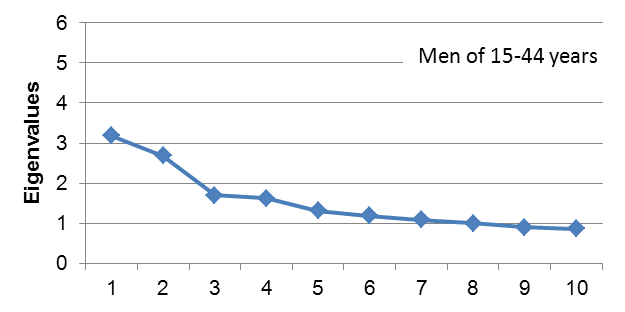

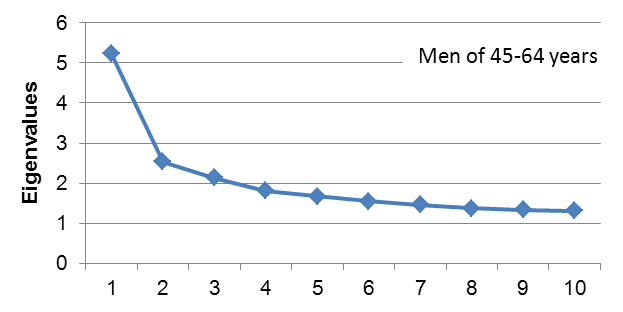

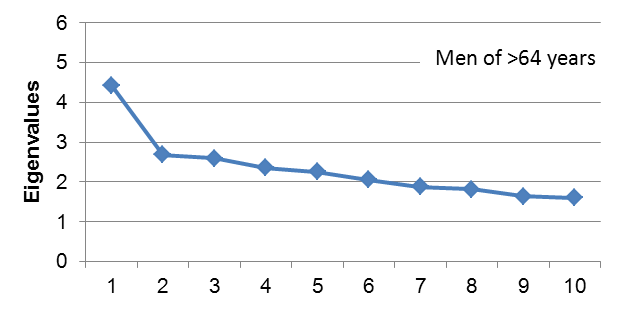

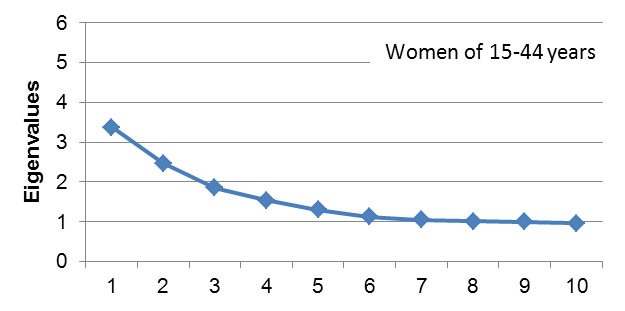

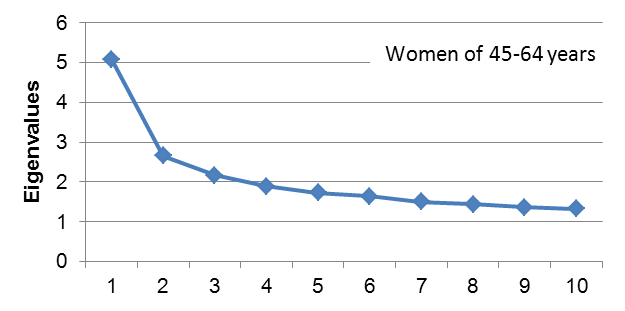

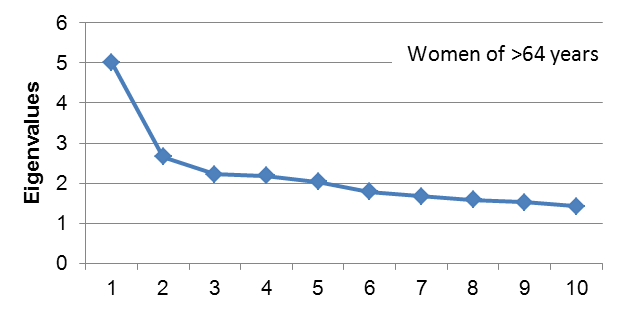

Supplement: File S1 — Combined file of supporting figures. Figure A - Scree plots for the different age and sex groups in the Spanish setting. Figure B - Scree plots for the different age and sex groups in the Dutch setting. (DOC) [file pone.0100375.s001.doc]
